# Higher Dimensional Meta-State Analysis Reveals Reduced Resting fMRI Connectivity Dynamism in Schizophrenia Patients

**DOI:** 10.1371/journal.pone.0149849

**Published:** 2016-03-16

**Authors:** Robyn L. Miller, Maziar Yaesoubi, Jessica A. Turner, Daniel Mathalon, Adrian Preda, Godfrey Pearlson, Tulay Adali, Vince D. Calhoun

**Affiliations:** 1 The Mind Research Network, Albuquerque, New Mexico, United States of America; 2 Department of Electrical and Computer Engineering, University of New Mexico, Albuquerque, New Mexico, United States of America; 3 Department of Psychology and Neuroscience, Georgia State University, Atlanta, Georgia, United States of America; 4 Department of Psychiatry, University of California San Francisco School of Medicine, San Francisco, California, United States of America; 5 Department of Psychiatry and Human Behavior, University of California Irvine School of Medicine, Irvine, California, United States of America; 6 Department of Psychiatry, Yale University School of Medicine, New Haven, Connecticut, United States of America; 7 Olin Neuropyschiatry Research Center, New Haven, Connecticut, United States of America; 8 Department of Computer Science and Electrical Engineering, University of Maryland Baltimore County, Baltimore, Maryland, United States of America; Universiteit Gent, BELGIUM

## Abstract

Resting-state functional brain imaging studies of network connectivity have long assumed that functional connections are stationary on the timescale of a typical scan. Interest in moving beyond this simplifying assumption has emerged only recently. The great hope is that training the right lens on time-varying properties of whole-brain network connectivity will shed additional light on previously concealed brain activation patterns characteristic of serious neurological or psychiatric disorders. We present evidence that multiple explicitly dynamical properties of time-varying whole-brain network connectivity are strongly associated with schizophrenia, a complex mental illness whose symptomatic presentation can vary enormously across subjects. As with so much brain-imaging research, a central challenge for dynamic network connectivity lies in determining transformations of the data that both reduce its dimensionality and expose features that are strongly predictive of important population characteristics. Our paper introduces an elegant, simple method of reducing and organizing data around which a large constellation of mutually informative and intuitive dynamical analyses can be performed. This framework combines a discrete multidimensional data-driven representation of connectivity space with four core dynamism measures computed from large-scale properties of each subject’s trajectory, ie., properties not identifiable with any specific moment in time and therefore reasonable to employ in settings lacking inter-subject time-alignment, such as resting-state functional imaging studies. Our analysis exposes pronounced differences between schizophrenia patients (N_sz_ = 151) and healthy controls (N_hc_ = 163). Time-varying whole-brain network connectivity patterns are found to be markedly less dynamically active in schizophrenia patients, an effect that is even more pronounced in patients with high levels of hallucinatory behavior. To the best of our knowledge this is the first demonstration that high-level dynamic properties of whole-brain connectivity, generic enough to be commensurable under many decompositions of time-varying connectivity data, exhibit robust and systematic differences between schizophrenia patients and healthy controls.

## Introduction

**Fig 1:**
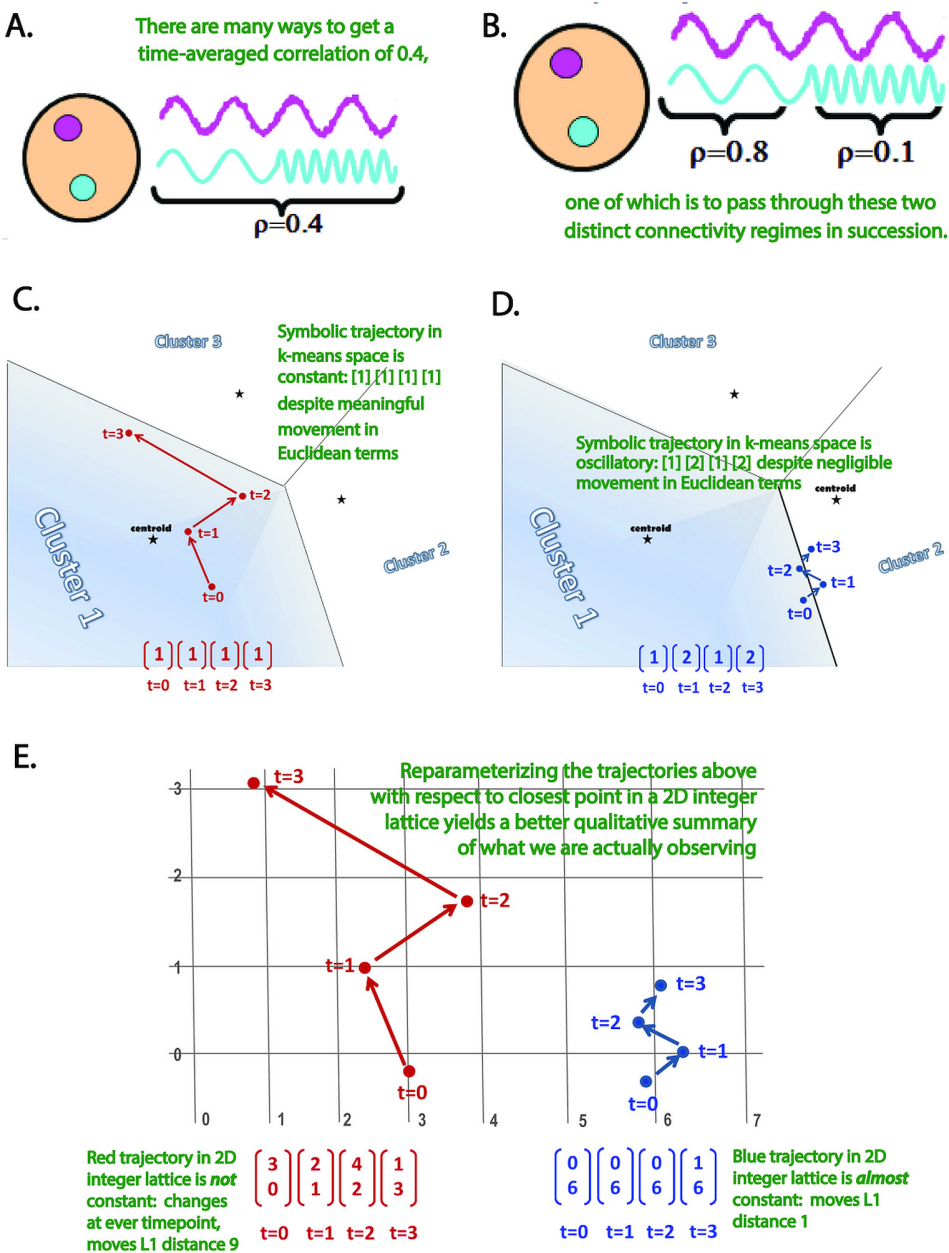
**Dynamic Connectivity, Single and Higher Dimensional Representations** (A) Example of two network timecourses whose correlation evaluated over their entire duration is 0.4; (B) One of the many different ways that a pair of long timecourses can have correlation coefficient of 0.4 is to pass through the two distinct, identifiable connectivity regimes shown here. The existence of the two connectivity regimes and the transitions between them are completely obscured by looking at correlation on a longer timescale; (C and D) Too crude a dimensionality-reduction of the state space can create serious distortions of the dynamics being analyzed. Dynamically active and mobile trajectories (C) can appear constant under the reduction, while those barely moving from their starting position (D) can seem highly dynamic; (E) Shifting up one dimension and characterizing the same trajectories by vectors reflecting their position In a discrete 2-dimensional state space yields much better qualitative agreement between the geometric trajectories and their symbolic representations.

Many neurological, cognitive and psychiatric disorders have been shown to affect connectivity between functional brain networks [1–24] even in so-called "resting" conditions where subjects are not engaged in a task. Network connectivity is typically assessed as a stationary feature of the data, inferred from the correlation or mutual information between pairs of network activation timecourses that extend through the duration of the scan. Although a useful simplification, there is no a priori reason to believe that network correlations are stationary, especially in the resting brain. In fact, one might expect cross-network connections to vary and evolve as subjects experience different thoughts, degrees of drowsiness, memories and emotional states. Far from being canonical, scan duration is simply one of the unavoidably fixed features of any functional imaging study. Thus, averaging evidence of connectivity over an entire resting fMRI scan puts researchers at risk of obscuring distinct, meaningful connectivity regimes that subjects are passing through (Fig 1A and 1B). Recent investigations of dynamic connectivity have in fact shown not only that connections are varying through time [25–36], but that this variation takes different forms in different demographic [35] and diagnostic [16, 26, 30, 32, 33, 37–39] groups

Most work on *dynamic functional network connectivity* (dFNC) to date has been focused on computing and statistically summarizing cross-network correlations evaluated separately on successive sliding windows through the original scan-length network timecourses [16, 25, 26, 30, 32, 33, 35, 37, 38, 40, 41]. The resulting window-indexed correlation matrices, called *windowed functional network connectivity* matrices (wFNC), record snapshots of network connectivity evolving in time. The collection of wFNCs for a given subject yields $\frac{N(N-1)}{2}$ length-*T* timeseries, one for each of network-pair correlation, where *T* is the number of windows and *N* the number of networks. The very first investigations [25, 26] of dynamic FNC used clustering as a dimensionality reduction tool, collapsing a $d=\frac{N(N-1)}{N}>1000$ dimensional connectivity space to just one dimension (ie., replacing an over 1000-dimensional object with the index *i* ∈ {1,2,…,*k*} of the cluster to which it belongs). Although some interesting results have emerged from this initial work, collapsing connectivity space onto a single dimension is the crudest possible dimension-reduction. A reduction of this magnitude inevitably obscures and distorts important features of dynamical network-coupling behavior (Fig 1C, 1D and 1E) that might characterize clinically or demographically defined groups

**Fig 2:**
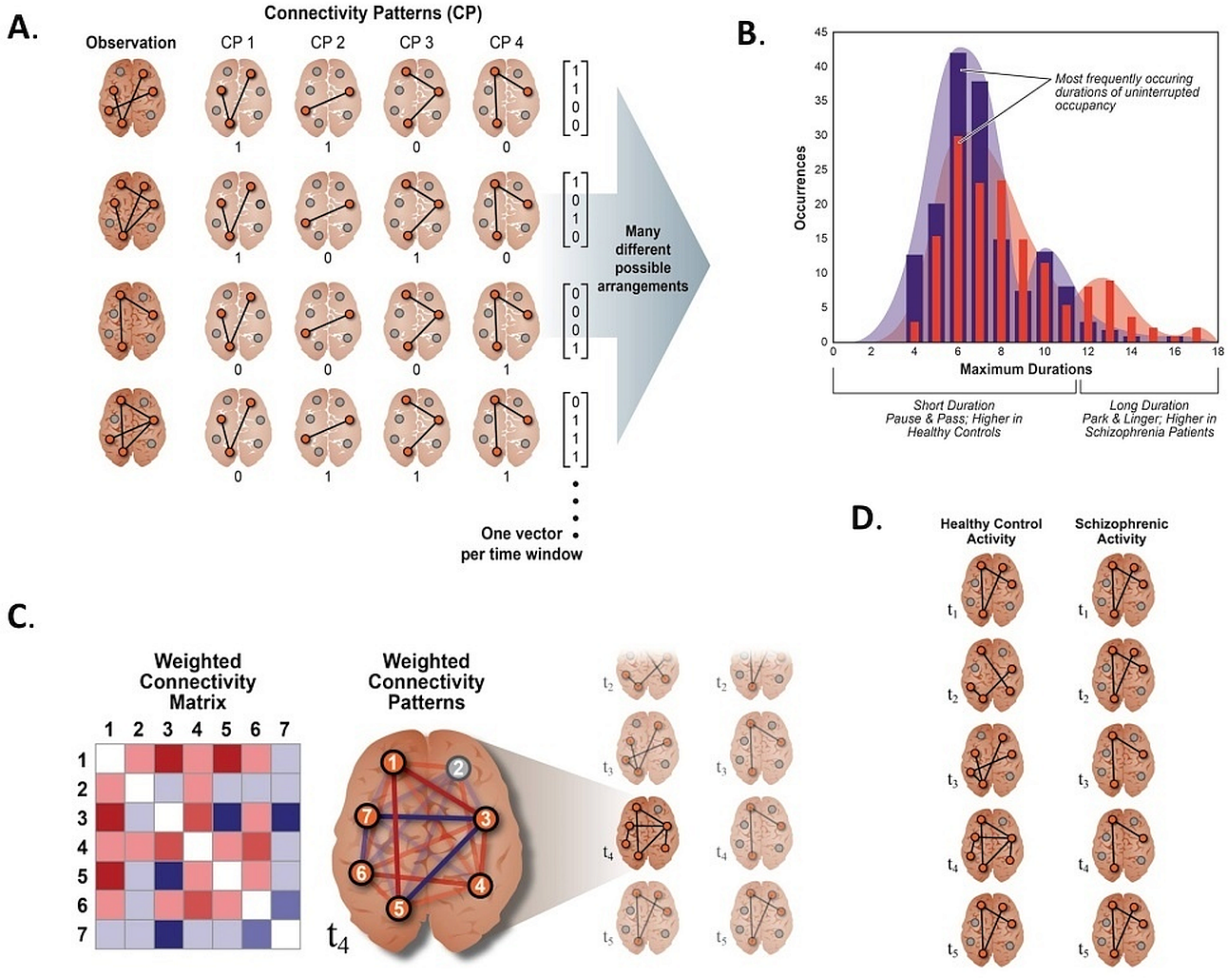
**Meta-State Dynamic FNC: High Level Schematics** (A) Schematic showing connectivity decomposed into, for simplicity, binary weighted sums of connectivity patterns, yielding meta-states in {0,1}s^4^; (B) Histograms of maximal uninterrupted periods spent in any fixed meta-state for patients (red) and controls (blue) (C) A connectivity pattern in which network-pair connections are signed and non-binary (for example, given by correlations) and in graph (right) and matrix (left) forms; (D) Schematic illustration of differences in dynamical patterns of network connectivity between schizophrenia patients and healthy controls. Controls exhibit more, and more diverse, connectivity states changing from one connectivity pattern to another more often than patients.

**Fig 3:**
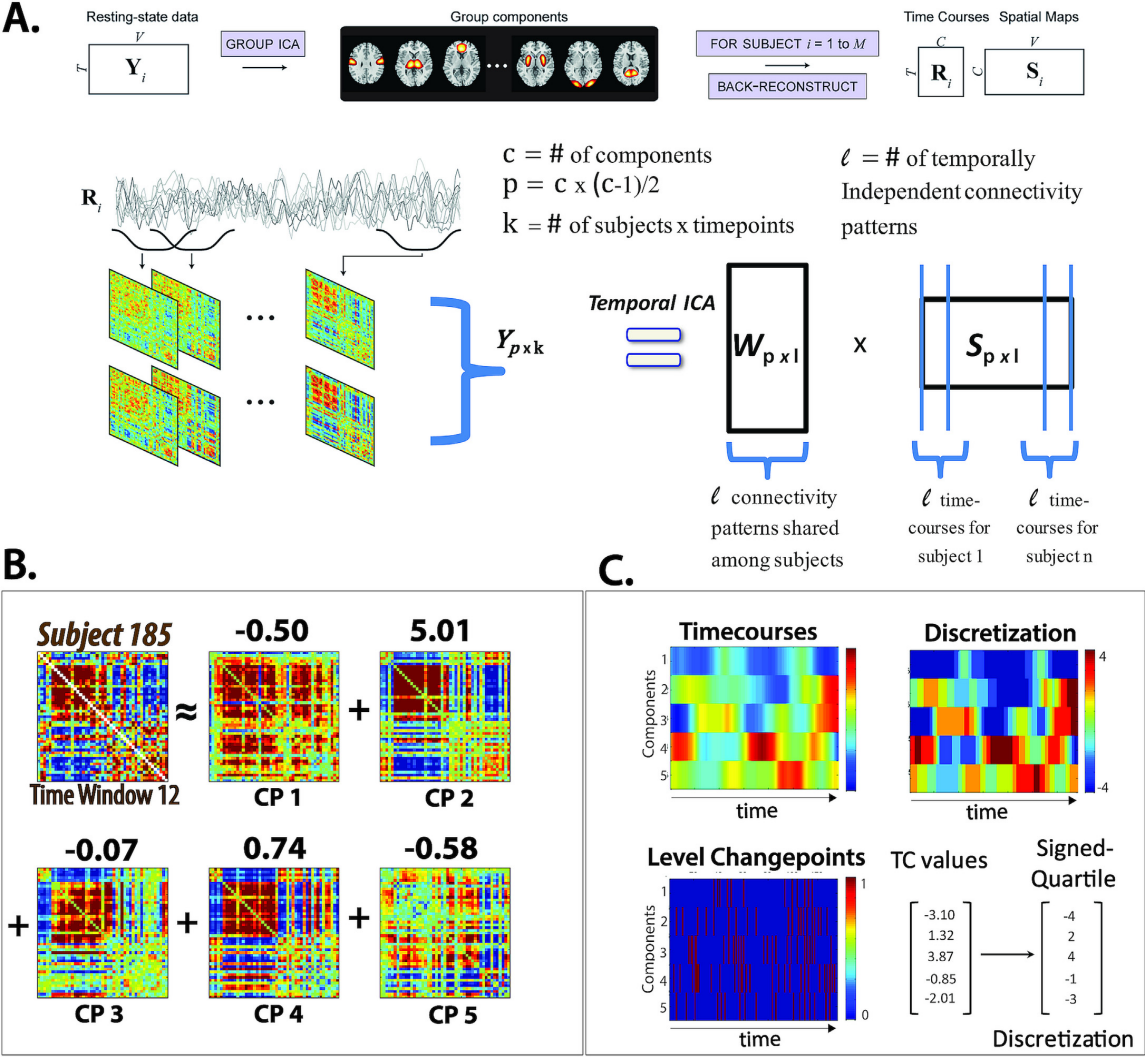
**Temporal ICA Schematic with Examples of Single Window Weighted CPs and Discretized Time-Varying CP Weights** (A) Schematic displaying stages involved in producing CPs; top row illustrates the initial decomposition of fMRI data into network spatial maps and corresponding timecourses using group spatial ICA (GICA); bottom row shows decomposition of window-indexed correlation matrices computed on sliding windows through the network timecourses (left-hand side of equation) produced by spatial ICA on fMRI data summarized in the top row into temporally independent CPs (matrix W on right hand side of equation) using temporal ICA; (B) Example of an observed wFNC expressed as weighted sum of the five displayed tICA CPs; (C) One subject's CP timecourses (top left) transformed into the signed quartile discretization (top right) with times at which each discretized timecourse changes from one level to another (bottom and example of one time-indexed 5-vector of timecourse values converted into a *meta-state* of signed quartile values (bottom right).

Our approach models windowed FNCs as weighted sums of maximally independent connectivity patterns (CPs) (Fig 2C and 2D), Fig 3A and 3B). Each wFNC is recast as a discretized vector of CP weights, called a *meta-state* (Fig 2(A), Fig 3(C)). This specific approach was motivated by a desire to understand network connectivity dynamics in terms of (not necessarily observable) patterns of signed network pair correlations that “pipe in” and fade out of observed wFNCs in a relatively independent manner. We introduce a set of simple dynamism measures easily calculated from subject trajectories through the induced discrete five-dimensional state-space, finding consistent, significant and replicable differences in connectivity dynamics between schizophrenia patients and healthy controls (Fig 2B and 2D). While the temporal behavior of specific network-pair correlations might be of interest in certain narrowly tailored questions, it seems natural to address complex brain diseases that encompass diverse categories and combinations of symptoms at a more aggregated level, examining how patterns or aggregates of network-pair correlations evolve *en masse* in afflicted populations. Schizophrenia is such a disease, and at the whole-brain level, we find very robust evidence of reduced dynamic fluidity and range in network correlation structure for patients suffering from this varied and complex disorder. The simultaneous weighted contributions, called *meta-states*, of whole-brain patterns of connectivity to subject wFNCs change less often, and shift between a smaller number of more similar meta-states in schizophrenia patients than in healthy controls (Fig 2(D)). Supporting a meta-state approach is the fact that this finding holds for sets of whole-brain network connectivity patterns, generally quite different from each other, derived using various data-driven approaches, including temporal independent component analysis (ICA), spatial ICA, principal component analysis (PCA) and k-means.

## Methods and Materials

### Sample and Data Acquisition

Resting state functional magnetic resonance imaging data (162 volumes of echo planar imaging BOLD fMRI, TR = 2 sec.) [26, 37] was collected from 163 healthy controls (117 males, 46 females; mean age 36.9) and 151 age and gender matched patients with schizophrenia (114 males, 37 females; mean age 37.8) during eyes closed condition at 7 different sites across the United States (Table 1). Inclusion criteria for the patients were a schizophrenia diagnosis based on the structured clinical interview for DSM-IV-TR axis I disorders (SCID-I/P) [42]. All patients were clinically stable on antipsychotic medication for at least 2 months, and had an illness duration of minimally one year. Clinical assessments for the patients included the positive and negative syndrome scale (PANSS) [43]. Written informed consent was obtained from all study participants, including permission to share de-identified data between the centers and with the wider research community. This analysis of existing data was approved by the institutional review board at the University of New Mexico.

**Table 1:** Demographic Information.

Subject Demographic Information			
Schizophrenia Patient (SZ)	151	Healthy Control (HC)	162
Male	231 (SZ = 114)	Female	83 (SZ = 37)
Ages 18–30	108 (SZ = 52)	Ages 31–60	206 (SZ = 99)

### Data Preprocessing

A combination of toolboxes (AFNI1, SPM2, GIFT3) and custom code written in Matlab were employed in the pre-processing pipeline. Rigid body motion correction was performed with the INRIAlign [44] toolbox in SPM to correct for subject head motion, followed by slice-timing correction to account for timing differences in slice acquisition. Data was then despiked using AFNI3s 3dDespike algorithm to mitigate the impact of outliers and despiked fMRI data was subsequently warped to a Montreal Neurological Institute (MNI) template, then resampled to 3 mm3 isotropic voxels. Instead of Gaussian smoothing, we smoothed the data to 6 mm full width at half maximum (FWHM) using AFNI3s BlurToFWHM algorithm which performs smoothing by a conservative finite difference approximation to the diffusion equation. This approach has been shown to reduce scanner specific variability in smoothness providing “smoothness equivalence” to data across sites [45]. Finally, prior to performing group independent component analysis, each voxel time course was variance normalized as this approach has been shown to yield better decompositions of subcortical and cortical sources.

### Decomposition into Functional Networks

After preprocessing, the functional imaging data from all subjects was decomposed into a set of 100 statistically independent spatial regions with common time course profile using group independent component analysis (GICA), implemented by the GIFT toolbox (http://mialab.mrn.org/software/gift). Of these 100 components, 47 were identified as intrinsic connectivity networks (ICNs) using the procedures described in our earlier work [26, 37]. Subject-specific spatial maps and time courses were obtained using spatio-temporal regression. The subject ICN time courses were detrended, orthogonalized with respect to motion parameters, despiked by replacing outlier time points with 3^rd^ order spline fit to cleaner neighboring points, and filtered using a 5^th^ order Butterworth filter with a passband of 0.01 to 0.15 Hz.

### Windowed Functional Network Connectivity Matrices (wFNCs)

Windowed functional network connectivity (wFNC) is evaluated by computing pairwise correlations between windowed segments of ICN timecourses using a tapered rectangular window of length of 22 TRs (44 seconds), advancing 1 TR at each step. To improve correlation estimates on timecourses of shorter length, we impose an L^1^ constraint on the inverse covariance matrix using the G-LASSO framework [45], with regularization parameter optimized subject-wise by evaluating the log-likelihood of each subject’s unseen data in a cross-validation framework.

### Basis Correlation Patterns (CPs)

Recent work [16, 25, 26, 37, 46] on functional network connectivity dynamics has used clustering algorithms to identify a small set of prototype connectivity “states”. Observed wFNCs are replaced by the prototype states they most resemble, allowing connectivity dynamics to be described as a process of moving from one to another of these summary states. In this work, we present a flexible, intuitive framework for studying network connectivity dynamics. The model order of five, used in all decompositions presented here, was chosen in an effort to balance tractability of complex linearly additive effects with a desire for richly featured basis correlation pattern sets. To assess the sensitivity of our results to perturbation of this parameter, we also performed our analysis on model orders ranging from three to seven, and found overall results to be consistent with the model order chosen as the focus of this paper, providing additional confidence in the robustness of the framework and the main dynamical metrics presented here.

#### Temporally Independent Connectivity Patterns

Our objective is to express time-varying wFNCs as weighted sums of correlation patterns whose contributions change independently of each other in time (see Fig 3(A) and 3(B)), allowing us to develop a richer picture of the interplay between connectivity patterns that are strongly present in the data. This objective explicitly permits collections of basis connectivity patterns featuring:

- Two or more patterns in which some subset of network-pairs share the same correlation strength.
- Individual patterns that do not strongly resemble empirically observed wFNCs.

To achieve this goal we apply group temporal independent component analysis (tICA) (Fig 3(A)) [30] to wFNC matrices concatenated along the subject×time dimension, decomposing this concatenated 1081 network-pair correlations × 136 time windows × 314 subject structure into five maximally mutually independent timecourses (because we are performing this analysis at the group level, these are in fact length 136∙314 = 42,704 subject×time “courses”), each with an associated 47×47 connectivity pattern (a modular component of the mixing matrix) that is shared across subjects (Fig 3(A)). In the text above, for convenience, we will refer to the connectivity patterns as components, even though it is the subject×timecourses that are being estimated by tICA. In this decomposition, individual wFNCs are specified as weighted sums of the five CPs, yielding a 5-dimensional characterization of each subject’s 1081-dimensional connectivity structure in each time window. The dynamical object of investigation is now a set of 136 time-indexed five-vectors per subject that representing the contributions of five 1081-dimensional CPs to the observed wFNCs. The tICA decomposition of wFNC data produces CPs whose weights in each time-indexed five-vector are maximally mutually independent [28–30]. These tICA CPs are thus patterns whose additive contributions to observed wFNCs “pipe in” and fade out in a relatively independent manner. Although the window-wise CP weights in the tICA decomposition are as independent as possible, intrinsic dependencies within the data ensure that the weights are not formally independent, i.e. $P(a_{1}\leq w_{1}\leq b_{1},\ldots ,a_{5}\leq w_{5}\leq b_{5})\neq \prod_{k=1}^{5}P(a_{k}\leq w_{k}\leq b_{k})$. The five-vectors thus hold information not available by analyzing elements separately, but maximizing temporal independence keeps the state-space from collapsing onto a lower-dimensional space, ie. if CP #1 and CP #2 are systematically mutually dependent then only one is necessary and the state space becomes four-dimensional. Reducing the systematic dependencies between CPs ensures we are taking maximal advantage of the dimensionality in which the dynamics have been defined.

Although we have chosen to focus on maximally temporally independent correlation patterns produced by applying temporal ICA to the windowed FNCs (Fig 3(A)), we were interested in understanding how sensitive the results obtained might be to our choice of method for extracting correlation patterns from the windowed FNCs. Thus we performed the same analysis on correlation patterns obtained from three other commonly utilized data-driven methods: spatial independent component analysis (sICA), principal component analysis (PCA) and kmeans clustering. We also explored the role of model order within the temporal ICA framework by repeating our analysis for temporal ICAs producing 2, 3, 6 and 7 (small perturbations of the featured 5 correlation pattern case).

#### Alternative Decompositions into CPs: Spatial ICA, Principal Components and K-means Cluster Centroids

**Fig 4:**
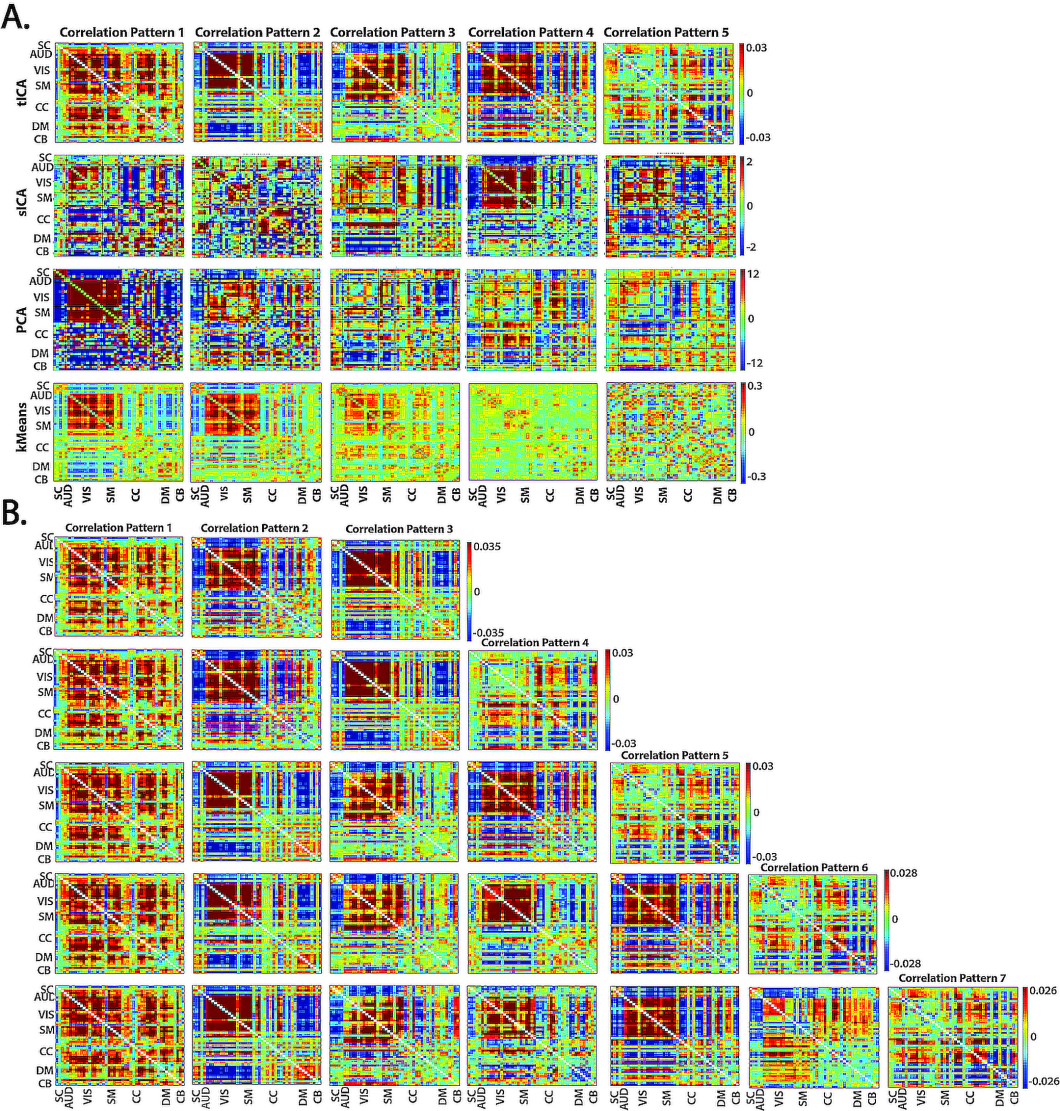
**Correlation Patterns Produced by Different Algorithms and by Temporal ICA at Different Model Orders** (A) Different data-driven decompositions of the wFNCs yield different sets of correlation patterns; correlation patterns produced, from top to bottom, by tICA, sICA, PCA, K-means, all with model order five; (B) Different model-orders of tICA applied to the wFNCs yield growing collections of correlation patters with two recurring patterns (Row 1, Columnss 1 and 3); tICA correlation patterns, from top to bottom, for model orders three through seven.

We perform a group (spatial) independent component analysis (GICA) on the wFNC data using protocols directly analogous those employed for higher-dimensional fMRI data [39, 43]. The basis correlation patterns obtained by group sICA are maximally spatially (cell-wise) independent, but neither mutually orthogonal nor informative about the way dFNC variance is organized (Fig 4 (A, Row 2)). For a set of mutually orthogonal basis patterns whose structure explicitly reflects dominant directions of data variance, we use the first five components of a PCA along the subject×time dimension of the concatenated wFNC data (Fig 4 (A, Row 3)). The timecourses for sICA (resp. PCA) correlation patterns are obtained by regressing each subject's wFNC data at each time window on the set of sICA (resp. PCA) correlation patterns.

In addition to ICA and PCA techniques, we also apply k-means clustering to the windowed FNCs. Using Matlab’s implementation of k-means clustering with the squared Euclidean distance, 500 iterates and 150 replicates, we partition the set of wFNCs into five clusters whose centroids are treated as basis correlation patterns (Fig 4 (A, Row 4)). We investigate the time-varying joint contributions of these CPs using two forms of *weighted k-means timecourses*. The first, directly analogous to the sICA and PCA timecourses, is a linearly additive weighting obtained by regressing wFNC data on the kmeans CPs. The second characterizes each wFNC by a five-vector with weights based on the L^2^ distances of the wFNCs from each kmeans CP. Specifically, for subject *k*'s time *t* wFNC, **F**^(*k*)^(*t*), we have the five vector $w^{\left(k\right)}(t)=(w_{1}^{\left(k\right)}(t),w_{2}^{\left(k\right)}(t),w_{3}^{\left(k\right)}(t),w_{4}^{\left(k\right)}(t),w_{5}^{\left(k\right)}(t))$ whose *i*^th^ element $w_{i}^{\left(k\right)}=1-\frac{\|F^{\left(k\right)}(t)-C_{i}{\|}_{2}}{\sum_{j=1}^{5}\|F^{\left(k\right)}(t)-C_{j}{\|}_{2}}$, where **C**_*j*_ is the *j*^th^ kmeans CP. For consistency with the other decompositions we report the results from regressing wFNC data on k-means CPs, but results using the alternative weighting system presented the same directionality and significance.

### Timecourse Discretization

We convert the original real-valued weight vectors to discrete *meta-states* (Fig 3(C)) by replacing each CP weight with a value in ±{1,2,3,4} according to its signed quartile: the vector $w^{\left(k\right)}(t)=(w_{1}^{\left(k\right)}(t),w_{2}^{\left(k\right)}(t),w_{3}^{\left(k\right)}(t),w_{4}^{\left(k\right)}(t),w_{5}^{\left(k\right)}(t))$ of subject *k*'s time *t* component weights is converted to $\lambda^{\left(k\right)}(t)=(\lambda_{1}^{\left(k\right)}(t),\lambda_{2}^{\left(k\right)}(t),\lambda_{3}^{\left(k\right)}(t),\lambda_{4}^{\left(k\right)}(t),\lambda_{5}^{\left(k\right)}(t))$ where $\lambda_{i}^{\left(k\right)}\in \{\pm 1,\pm 2,\pm 3,\pm 4\}$ indicating the quartile of the (same-sign) weights each w_i_^(k)^ falls into. When $\lambda_{i}^{\left(k\right)}=l\in \{\pm 1,\pm 2,\pm 3,\pm 4\}$, component *i* is said to be *occupied* at level l. The length-five vectors $\left(\lambda_{1}^{\left(k\right)}(t),\lambda_{2}^{\left(k\right)}(t),\lambda_{3}^{\left(k\right)}(t),\lambda_{4}^{\left(k\right)}(t),\lambda_{5}^{\left(k\right)}(t)\right)$ are referred to as *meta-states*. Discretization of sICA, principal component and weighted k-means timecourses follows the tICA procedure exactly.

2.9 Diagnosis Effects

We employ a linear model, *y* = *β*_0_ + *β*_*age*_*X*_*age*_ + *β*_*gender*_*X*_*gender*_ + *β*_*diagnosis*_*X*_*diagnosis*_ + *ε* to estimate the effect of diagnosis on the various measures investigated here. The diagnosis variable is binary, with SZ coded as '1' and HC as '0', so *β*_*diagnosis*_ > 0 indicates a positive correlation with SZ and *β*_*diagnosis*_ < 0 shows a negative correlation with SZ. We generally report or display the value of *β*_*diagnosis*_ when its false discovery rate (FDR) corrected p-value is less than 0.05.

## Results

Our meta-state dynamical framework exposes important features of whole-brain connectivity dynamics that seem to emerge specifically at this level of analysis; network timecourses [1], windowed network timecourses, and even individual network-pair wFNC values, for example, exhibit more high-frequency power in schizophrenia patients than in controls, a phenomenon that dissolves the whole brain meta-state level (see Discussion section). The persistence of our findings across a variety of approaches to identifying individual states from the data suggests we are observing robust phenomena at this particular scale of analysis. To gauge the robustness of our findings, we applied the meta-state analytical framework to tICA decompositions with smaller (model orders = 3, 4) and larger (model orders = 6, 7) sets of CPs, and also to collections of five CPs produced using group spatial ICA (GICA), PCA and k-means clustering (Fig 4(A)).

### Broad Reduction of Dynamic Fluidity and Range in Schizophrenia Patients

**Fig 5:**
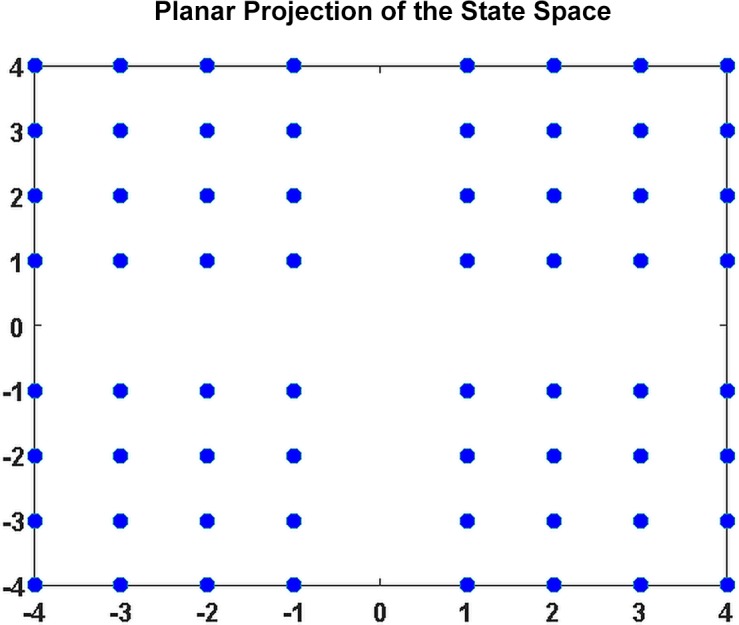
Planar projection of the discrete five-dimensional state space.

The state space **X** induced by our discretized five-dimensional characterization of connectivity is a five-dimensional lattice with a set of five mutually orthogonal co-dimension one hyperplanes through the origin removed (Fig 5).

Each removed hyperplane is the zero set of one of the dimensions (these hyperplanes are not part of **X** because the range of our signed-quartile discretization is {±1,2,3,4}. The space contains C ≡ 8^5^ = 32,768 distinct points (meta-states), of which c_R_ = 14,025 are realized at some point in time by some subject. Individual subjects can visit at most T = 136 meta-states, and our sample of N = 314 subjects allows for entire sample to visit at most NT = 42,704 meta-states. Although the entire study includes more state visits (42,704) than points in the **X**, more than half (C-c_R_ = 18,743 or 57.2%) of the points in **X** are never visited. The combinatorics make a full exploration of ways unvisited states distribute in **X** prohibitive, but some high-level facts about how individual subjects sample the state space are readily established. Our analysis focuses on four global metrics of connectivity dynamism:

1. The number of times that subjects *switch* from one meta-state to another (denoted by ***s***)
2. The *number* of distinct meta-states subjects occupy during their scans (denoted by ***n***)
3. The *range* of meta-states subjects occupy, ie., the largest L^1^ distance between occupied meta-states (denoted by ***r***)
4. The overall *distance* traveled by each subject through the state space (the sum of the L^1^ distances between successive meta-states, denoted by ***d***)

The first measure captures how often a subject switches between meta-states, without accounting for how many or how divergent the meta-states are (one could switch between two very similar states in rapid succession). The second records the number distinct meta-states are passed through. Since there are 32,768 distinct meta-states available, a very high ratio of ***n*** to the number of time points implies high ***s***; a very high ratio of ***n*** to the number of possible meta-states implies high ***r***. The third measure indicates how divergent the meta-states occupied are. The value of ***r***, except when identically zero, need not imply anything about ***s*** or ***n***. It is a lower bound for ***d***. The final measure, ***d***, incorporates information from the other three without being fully determined by them. It is maximized when a subject switches frequently between two meta-states at distal boundaries of the state space.

**Fig 6:**
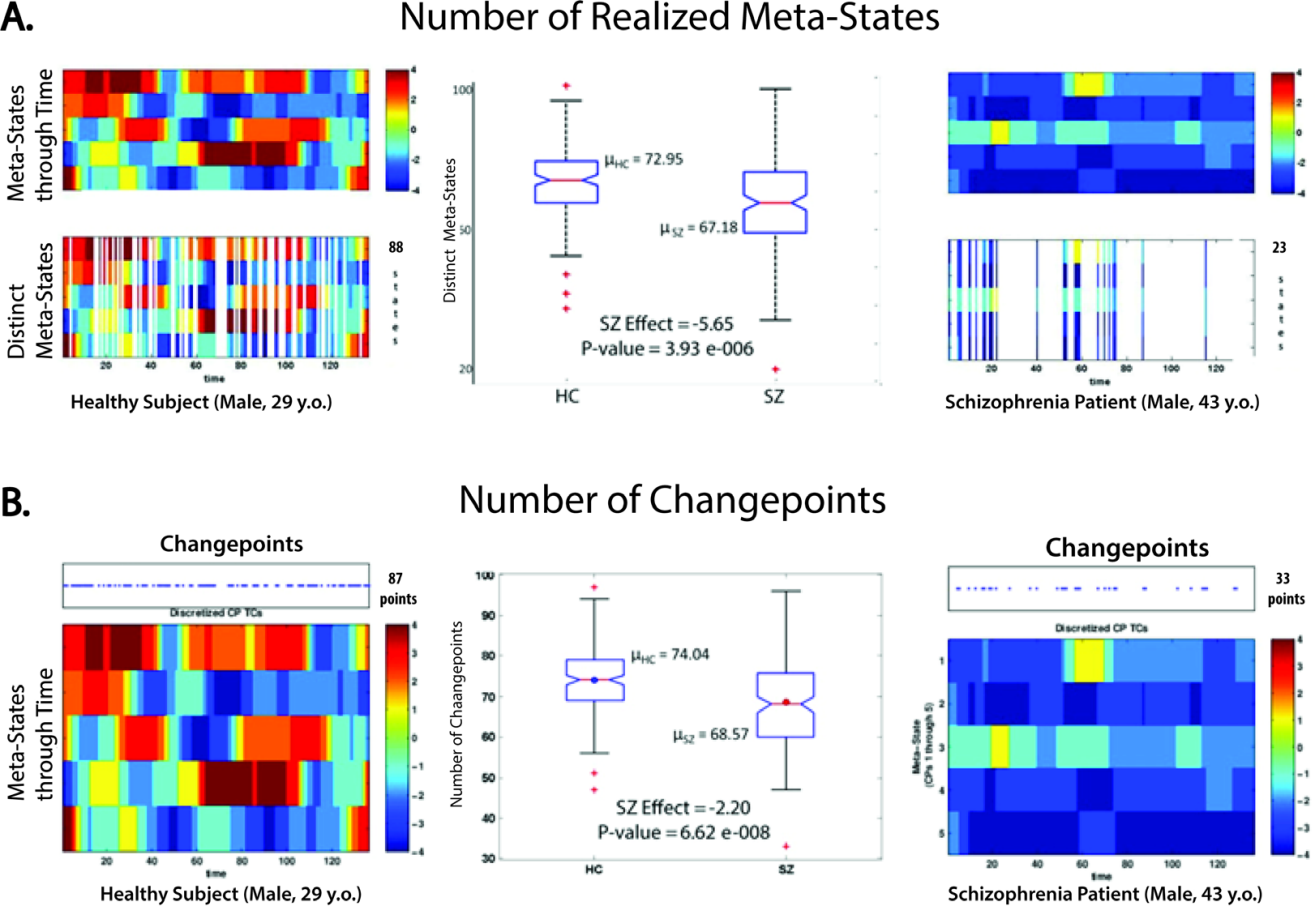
**Effect of Schizophrenia on Dynamic Fluidity Measures** (A) Number of meta-states realized; (middle column) boxplot showing median, quartiles and outliers plus mean for each group, and diagnosis effect from regression model specified in the Methods section with associated p-value; (leftmost column) The time-indexed meta-states of a sample healthy subject, with the 88 distinct meta-states shown underneath; (rightmost column) The time-indexed meta-states of a sample schizophrenia patient, with the 23 distinct meta-states shown underneath; (B) Number of timepoints at which subjects change between meta-states; (middle column) boxplot showing median, quartiles and outliers plus mean for each group, and diagnosis effect from regression model specified in the Methods section with associated p-value; (leftmost column) The time-indexed meta-states of a sample healthy subject, with the 87 timepoints at which meta-state changes shown above; (rightmost column) The time-indexed meta-states of a sample schizophrenia patient, with the 33 timepoints at which meta-state changes shown above.

**Fig 7:**
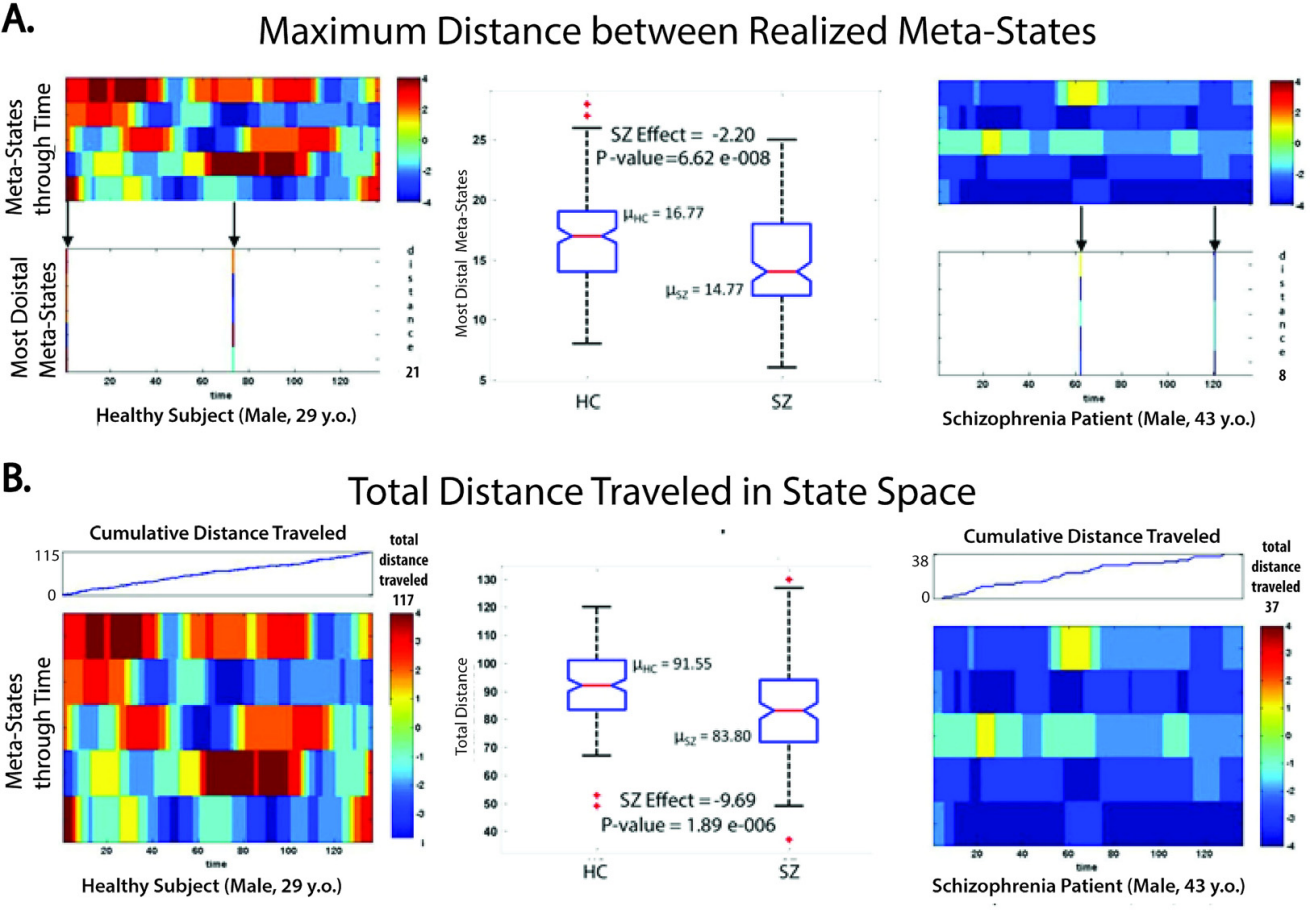
**Effect of Schizophrenia on Dynamic Range Measures** (A) Maximally different (in the L1 sense) meta-states that subjects realize; (middle column) boxplot showing median, quartiles and outliers plus mean for each group, and diagnosis effect from regression model specified in the Methods section with associated p-value; (leftmost column) The time-indexed meta-states of a sample healthy subject, with the two most divergent realized meta-states (L1 distance = 21) shown underneath; (rightmost column) The time-indexed meta-states of a sample schizophrenia patient, with the two most divergent realized meta-states (L1 distance = 8) shown underneath; (B) Total distance traveled (summed L1 distance between successive meta-states) in the state space; (middle column) boxplot showing median, quartiles and outliers plus mean for each group, and diagnosis effect from regression model specified in the Methods section with associated p-value; (leftmost column) The time-indexed meta-states of a sample healthy subject, with the timeseries of cumulative distance traveled (increasing to 117 at final timepoint) shown above; (rightmost column) The time-indexed meta-states of a sample schizophrenia patient, with timeseries of cumulative distance traveled (increasing to 37 at final timepoint) shown above.

We find consistent and pervasive evidence (Table 2) of reduced FNC dynamism among schizophrenia patients with respect to each of the four metrics above, and across all four data-driven decompositions of observed wFNCs into collections of basis CPs:

1. SZ exhibit diminished *dynamic fluidity*:
  - Schizophrenia patients occupy fewer meta-states than healthy controls (mean HC = 73.2 meta-states, mean SZ = 67.4 meta-states; diagnosis effect in regression = -5.65, P-value = 3.93 e-006). (Fig 6(A), Table 2, Table 3)
  - Schizophrenia patients change from one meta-state to another less often than healthy controls (mean HC = 74.04 changes, mean SZ = 68.58 changes; diagnosis effect in regression = -5.32, P-value = 1.72 e-006). (Fig 6(B), Table 2, Table 3)
2. SZ operate over a restricted *dynamic range*:
  - Schizophrenia patients remain trapped in a smaller radius hypercube of the state space than do healthy controls, as measured by the maximal L1-distance between occupied meta-states (mean HC = 16.77 diameter, mean SZ = 14.77 diameter; diagnosis effect in regression = -2.20, P-value = 6.62 e-008). (Fig 7(A), Table 2, Table 3)
  - Schizophrenia patients traverse less overall distance, evaluated by summed L1 distance between successive meta-states, through the state space than do healthy controls (mean HC = 91.55 overall distance, mean SZ = 83.80 overall distance; diagnosis effect in regression = -9.69, P-value = 1.89 e-006). (Fig 7(B), Table 2, Table 3).

**Table 2:** Age and gender-corrected effects of SZ on four general dynamism measures (rows) under four different decompositions of the wFNC data into sets of five correlation patterns (columns).

	Method of Decomposing wFNCs into CPs			
	tICA	sICA	PCA	K-means
**Number of Distinct Meta-States**	-5.65	-2.63	-5.78	-6.52
	p = 3.93e-006)	(p = 0.007)	(p = 6.03e-008)	(p = 1.52e-007)
**Number of Meta-State Changes**	-5.32	-2.59	-4.80	-5.57
	(p = 1.41e-008)	(p = 0.003)	(p = 5.88e-007)	(p = 8.23e-007)
**L1 Span of Realized Meta-States**	-2.20	-1.29	-2.22	-2.71
	(p = 6.62e-008)	(p = 2.71e-006)	(p = 2.09e-010)	(p = 1.52e-009)
**Total Distance Traveled in State Space**	-9.69	-4.29	-6.78	-10.04
	(p = 1.89e-006)	(p = 0.0009)	(p = 1.76e-006)	(p = 5.31e-007)

Displayed effects and p-values are from the regression model specified in the Methods section.

**Table 3:** Age and gender-corrected effects of SZ on four general dynamism measures (rows) computed over tICA decompositions of different model orders (columns). Displayed effects and p-values are from the regression model specified in the Methods section.

	Number of tICA CPs				
	3	4	*5*	6	7
**Number of Distinct**	-5.36	-6.28	***-5*.*65***	-5.45	-5.80
**Meta-States**	(p = 4.26e-006)	(p = 1.86e-007)	***(p = 4*.*11e-006)***	(p = 5.64e-007)	(p = 2.37e-009)
**Number of Meta-State**	-3.93	-5.06	***-2*.*20***	-5.15	-5.48
**Changes**	(p = 9.64e-005)	(p = 3.09e-006)	***(p = 6*.*62e-008)***	(p = 4.98e-007)	(p = 6.22e-009)
**L1 Span of Realized**	-1.24	-1.93	***-1*.*94***	-2.64	-3.07
**Meta-States**	(p = 7.33e-005)	(p = 1.52e-007)	***(p = 3*.*11e-005)***	(p = 2.62e-008)	(p = 1.65e-009)
**Total Distance Travel-**	-5.02	-7.50	***-7*.*53***	-11.04	-14.60
**ed in State Space**	(p = 2.68e-004)	(p = 8.47e-006)	***(p = 1*.*01e-005)***	(p = 1.06e-006)	(p = 1.37e-006)

**Fig 8:**
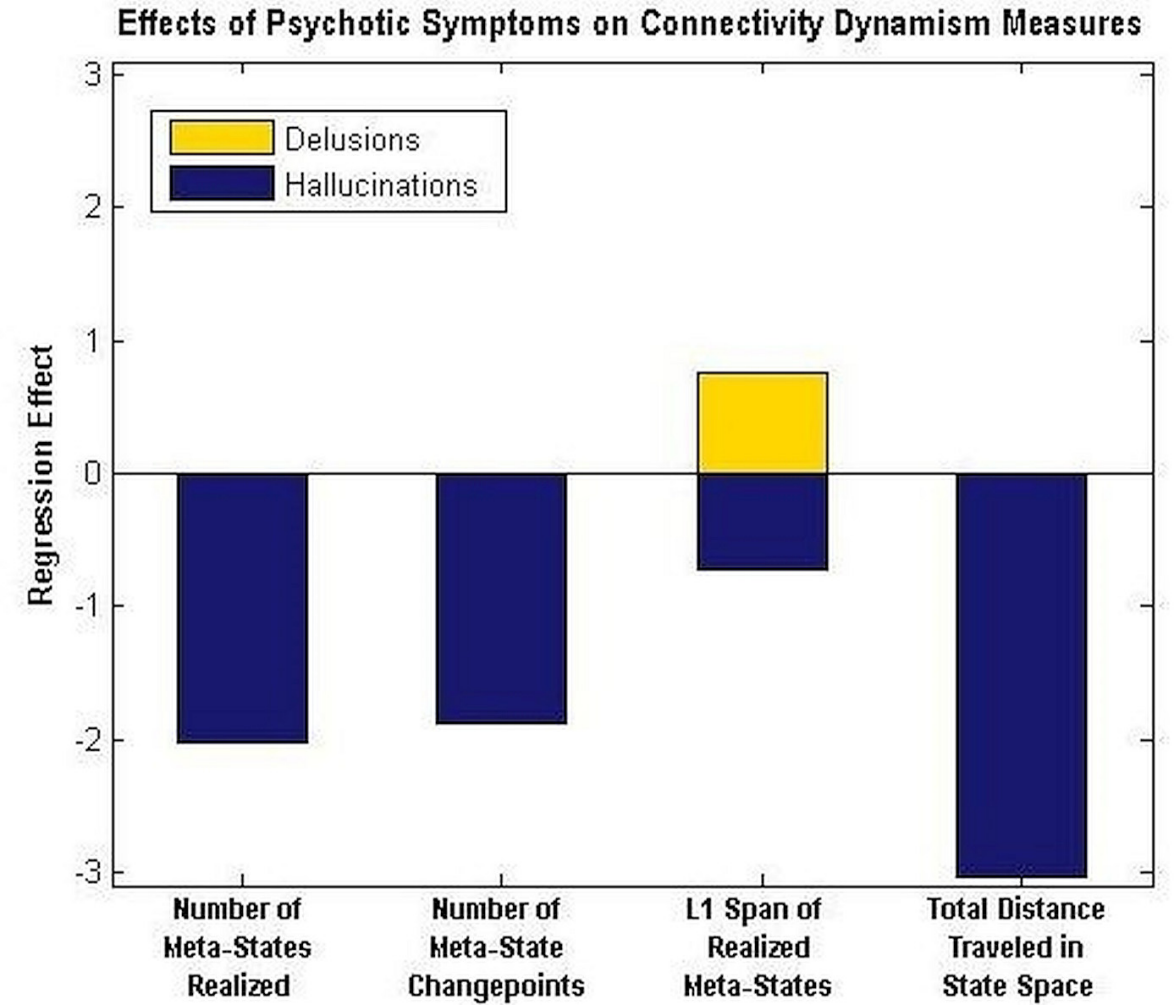
Effect of Main Psychotic Symptoms of Schizophrenia on Connectivity Dynamism Measures Significant (α<0.05) effects of hallmark psychotic symptoms of SZ on each of the four dynamism measures from regression on all thirty symptom scores from the PANSS scale along with gender and age as covariates. The effect of delusions on L1 Span of Realized Meta-States has p-value = 0.023. The p-values associated to the effects of hallucinatory behavior on each of the four measures given along the x-axis are, from left to right: 0.005, 0.003, 0.003, and 0.010.

Although schizophrenia patients exhibit diverse symptoms [43] ranging from blunted affect and withdrawal to grandiosity, the hallmark psychotic symptoms of the disease, delusions and hallucinations, are of special interest. Initial investigations of connectivity dynamism at the symptom level show mixed effects of the main psychotic underpinnings of schizophrenia with delusions having weakly dynamism-amplifying effects and hallucinations presenting significant and pervasive dynamism-suppressing effects (Fig 8).

### Schizophrenia Patients have more Hub States than Controls

**Fig 9:**
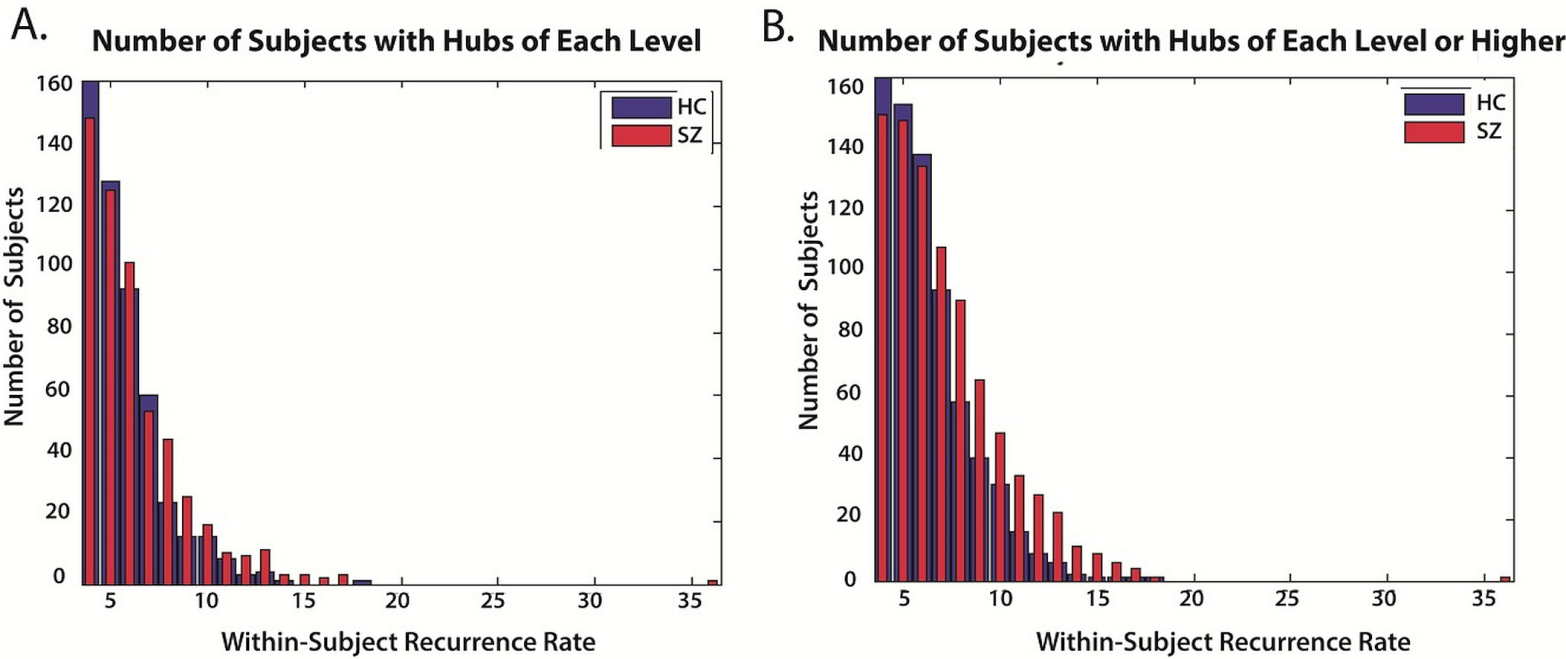
**Basic Results on Hub Meta-States and Schizophrenia** (A) Bar plots for HC and SZ of number of subjects (y-axis) with hubs of *exactly* the indicated levels (x-axis); (B) Bar plots for HC and SZ of number of subjects (y-axis) with hubs of *at least* the indicated level (x-axis).

**Fig 10:**
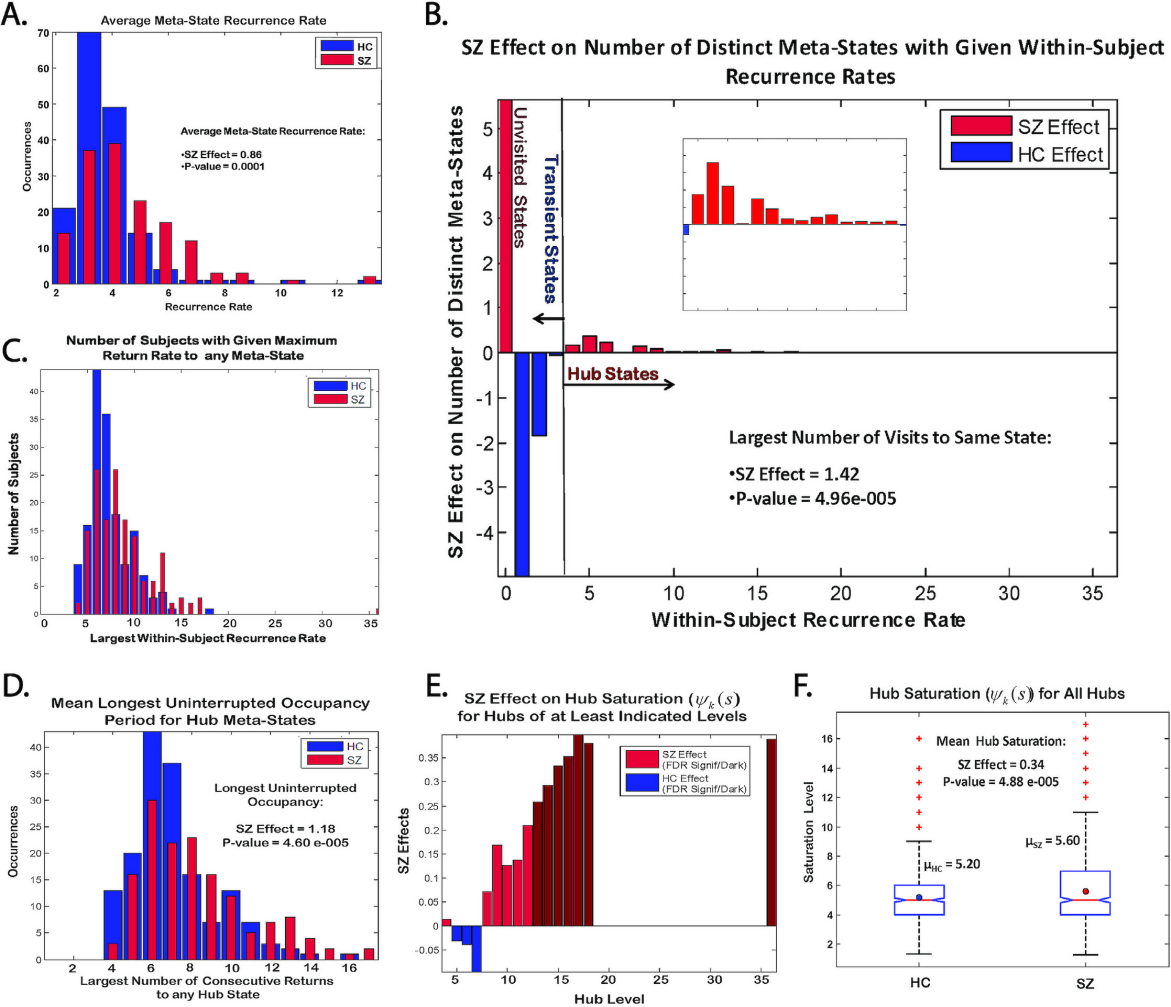
**Results of Comprehensive Investigation of Hub Meta-States and Schizophrenia** (A) Histograms of within-subject mean meta-state recurrence rate (average number of re-visitations made to the meta-states realized) and SZ regression effect on this quantity (SZ effect = 0.86, p-value = 0.0001); (B) SZ effect on number of distinct meta-states with indicated within-subject recurrence rate (inset zooms on rates 3–18) along with SZ effect on the largest number of visits to the same state (SZ effect = 1.42, p-value = 4.96e-005 (also see (C) for distribution)); (C) Number of subjects whose most visited meta-state had indicated recurrence rate; (D) Mean longest *uninterrupted* period of hub state occupancy (recall that level k hubs are occupied k times, not necessarily in uninterrupted stretches) and SZ regression effect (SZ effect = 1.18, p-value = 4.60e-005); (E) Effect of SZ on hub saturation, evaluated separately for hubs of each level k, k = 4,5,…,36. Saturation positively correlated with SZ in red (dark red indicates effects significant 0.05 level after FDR correction), negatively correlated with saturation in blue. (F) Boxplot of saturation index over all hubs for HC and SZ with group means (μ_SZ_ = 5.2, μ_HC_ = 5.6) and SZ regression effect on this quantity (SZ effect = 0.34, p-value = 4.88e-005); All SZ effects and associated p-values are from the regression model specified in the Methods section. The reported regressions include the patient with an order-36 hub (ie, they are for the displayed data, and we did want to provide evidence that hubs of higher order are achievable). In regressions omitting this extreme observation, both the direction and significance level of displayed effects were preserved (p-values were on order e-005 or e-006).

Although healthy subjects on average pass through a significantly larger number of distinct connective meta-states during their resting fMRI scans (Fig 6(A)), it is patients that include a larger set of distinct meta-states among those they visit more than three times. More concretely, healthy subjects visit a significantly larger number of distinct meta-states at least once, while patients visit a significantly larger number of distinct meta-states at least four times (Fig 9, Fig 10A, 10B and 10C)). The overall dominance of healthy controls is taking place entirely via the number of different "filler" meta-states they touch on briefly while passing between meta-states they visit more often.

**Fig 11:**
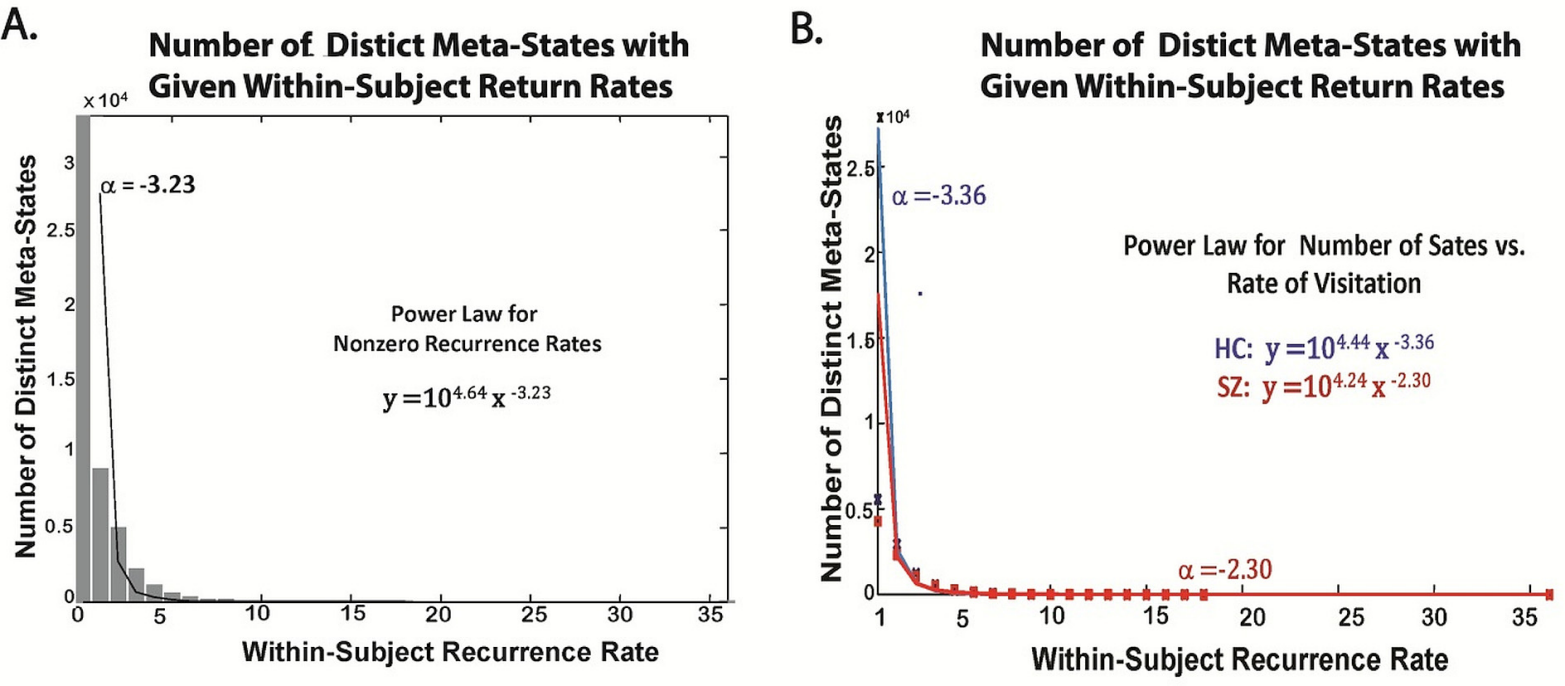
**Number of Distinct Meta-States Occupied Multiple Times by any Subject** (A) Bar plot of number of meta-states with indicated within-subject recurrence rates for full population with fitted power law (α = -323); (B) Power laws fitted separately for HC(α = -3.36) and SZ (α = -2.30) to number of meta-states with given within-subject recurrence rates.

The meta-states that a subject returns to four or more times are the subject's *hub* states; those that the subject visits at least once but no more than three times are the subject's *transient* states. A state that is visited *k*≥4 times is called a *level-k hub*. In our data, the complete set of level-*k* hubs for *k≥*4 consists of 2117 meta-states, accounting for approximately 6% of whole state space (C = 32,768) space and 15% of the points that are ever visited at least once by some subject (c_R_ = 14,025) (Fig 11). Every subject in this study has a level-*k* hub for *k* = 4 (Fig 9(A)) and only one subject has a level-*k* hub for *k*>18 (Fig 9(B)). Not only do patients exhibit a larger number of distinct hub meta-states during their scans (Fig 10A and 10B), Fig 11), they tend to spend longer *uninterrupted* periods than controls occupying these meta-states (Fig 10C, 10D and 10E)). A patient, for example, with some level-20 hub is more likely to spend three uninterrupted time intervals of lengths 5, 7 and 8 in that meta-state, while a healthy subject is more likely to spend eight uninterrupted intervals of lengths 2, 1, 4, 1, 2, 1, 6 and 3 in one of its level-20 hub states. A subject whose longest uninterrupted occupancy of a level-*k* hub is a large relative to *k* is said to *saturate* that hub; level-*k* hubs whose longest uninterrupted periods of occupancy are large relative to *k* are *absorbing hubs*. The level-*k* hubs that subjects land on *k* times but always move on from quickly are *transitory hubs*. Patients have more hubs than controls, and these hubs are more absorbing (Fig 10D, 10E and 10F)).

Although there are multiple supportable ways to quantify the degree to which level-*k* hubs are absorbing or saturated, we have chosen to employ the following within-subject measure of level-*k* saturation:

$$\psi_{k}(s)=\frac{\left(longest\;uninterrupted\;occupancy\;of\;a\;level\;k\;hub)(\#\;of\;times\;the\;maximal\;level\;k\;occupancy\;occurs\right)}{max(1,(\#of\;level\;k\;hubs))}$$

$\psi_{k}(s)=\frac{kd}{max(1,d)}=k$ when subject *s* has *d* level-*k* hubs, each of which the subject spends *k* consecutive time points in. Otherwise, $\psi_{k}(s)=\frac{k^{\prime }d^{\prime }}{max(1,d)}\leq k$, where *k'≤k* is the maximal uninterrupted occupancy of one of the subject's *d* level-*k* hubs and *d'≤d* is the number level-*k* hubs that the subject occupies for an uninterrupted period of length *k'*. The idea is to capture, in a way that gives more "credit" for saturations of higher-level hubs, the extent to which a subject utilizes the available visits to his or her level-*k* hubs in uninterrupted occupation.

The number of distinct meta-states visited exactly *n* times by some subject decays exponentially with recurrence rate (Fig 11). The rate of decay is slower for patients than controls (Fig 11(B)). The number of subjects returning to any meta-state at least *m* times, follows a roughly right-skewed normal distribution with respect to the maximum return rate *m* (Fig 10(C)). Every subject has at least one hub, ie. they visit some meta-state four or more times. Only eleven subjects (3% of the total), however, visit no state *more than* four times (Fig 10(C)). The average maximum return rate is 8.4 for patients and 7.2 for controls. Fewer than 10% of subjects return to any state more than eleven times (Fig 10(C)). And in terms of the state space, just over 0.1% of states are visited more than eleven times by some subject (Fig 10(B), Fig 11). It is also the case that patients move significantly more smoothly through the state space than do healthy controls, ie. the average L^1^ distance between successive meta-states is smaller in the patient population (SZ Effect = -0.08, P-value = 3.04 e-008).

### Trajectories of Groups of Schizophrenia Patients More Likely to Intersect at Common Meta-Sates than are Trajectories of Healthy Controls

**Fig 12:**
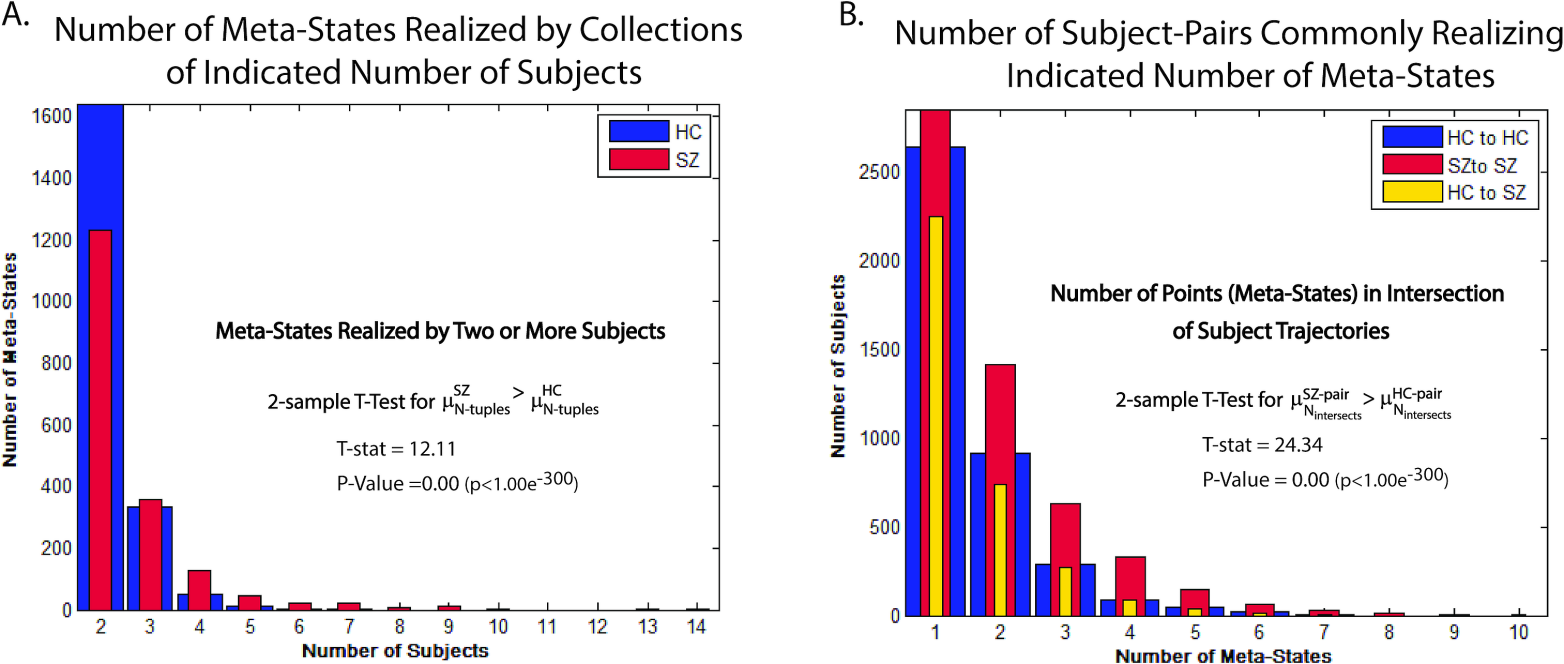
**Meta-States Realized at Least Once by Multiple Subjects** (A) Bar plot of number of meta-states realized by two or more subjects (number of subjects in the collection jointly realizing some meta-state on the x-axis), shown separately for patients and healthy controls. No meta-state whatsoever is realized by more than 7 different healthy subjects. Fewer than 50 (of 32,768) meta-states are realized by more than 6 patients. A two-sample T-test shows that the number N_SZ_ of patients jointly realizing some meta-state is significantly larger than the number N_HC_ of controls respectively jointly realizing some meta-state; (B) Bar plot of the number of subject-pairs that jointly realize indicated (x-axis) number of meta-states, shown separately for pairs of controls, pairs of patients and mixed pairs consisting of one patient and one control. A two-sample T-text shows that the average number of intersection points (jointly realized meta-states) for SZ subject pairs is significantly larger than the average number of intersection points for HC subject pairs.

As noted above, the discrete 5-dimensional state space in which we are studying dynamics is large relative to the length of the trajectories (the state space **X** contains 32,768 points while trajectories consist of only 136 observed timepoints). Absence of inter-subject temporal alignment in resting fMRI means that most subjects start at some arbitrary point in the state space, move around for 136 timepoints in a manner at least moderately constrained by their arbitrary “initial condition”, and need not intersect any other subject trajectories at all. For schizophrenia patients this problem is more pronounced since they realize fewer meta-states, change meta-state less often and remain trapped in a smaller radial neighborhood of their arbitrary starting point. This makes it all the more interesting to note that pairs of schizophrenia patients are significantly more likely to have trajectories that intersect than are healthy controls. They are in fact more likely than pairs of controls to have trajectories that intersect up to ten times (Fig 12(B)). The odds that the trajectory of a patient and that of a control intersect are lower still (Fig 12(B)). It is also the case that the number of distinct meta-states (points in **X**) realized by more than a handful of subjects, i.e., for which there exist three or more subjects whose trajectories intersect at that point is very small (1751, or about ½ of 1% of the points in **X**). Although individual schizophrenia patients realize fewer meta-states than do healthy controls, it is also the case that a significantly larger number of distinct meta-states are realized by collections of three or more schizophrenia patients than by groups of healthy controls (Fig 12(A)).

## Discussion

We have introduced a flexible, robust framework for analyzing dynamic network connectivity in terms of a small set of whole-brain correlation patterns that combine additively to form (or approximate) observed time-varying windowed FNCs, and whose simultaneous contributions, in magnitude and direction, are maximally mutually independent. We investigate the temporally-indexed sequence of length-five CP weight vectors, or meta-states. At this level, we find very strong statistical evidence that:

1. SZ exhibit diminished *dynamic fluidity*:
  - SZ switch less frequently between five-dimensional meta-states (Fig 6, Table 2, Table 3)
  - SZ occupy a smaller number of distinct meta-states during their scans (Fig 6, Table 2, Table 3)
2. SZ operate over a restricted in *dynamic range*:
  - SZ remain trapped in a smaller radius hypercube of the state space (Fig 7, Table 2, Table 3)
  - SZs cover less distance as they move through the state space (Fig 7, Table 2, Table 3)

**Fig 13:**
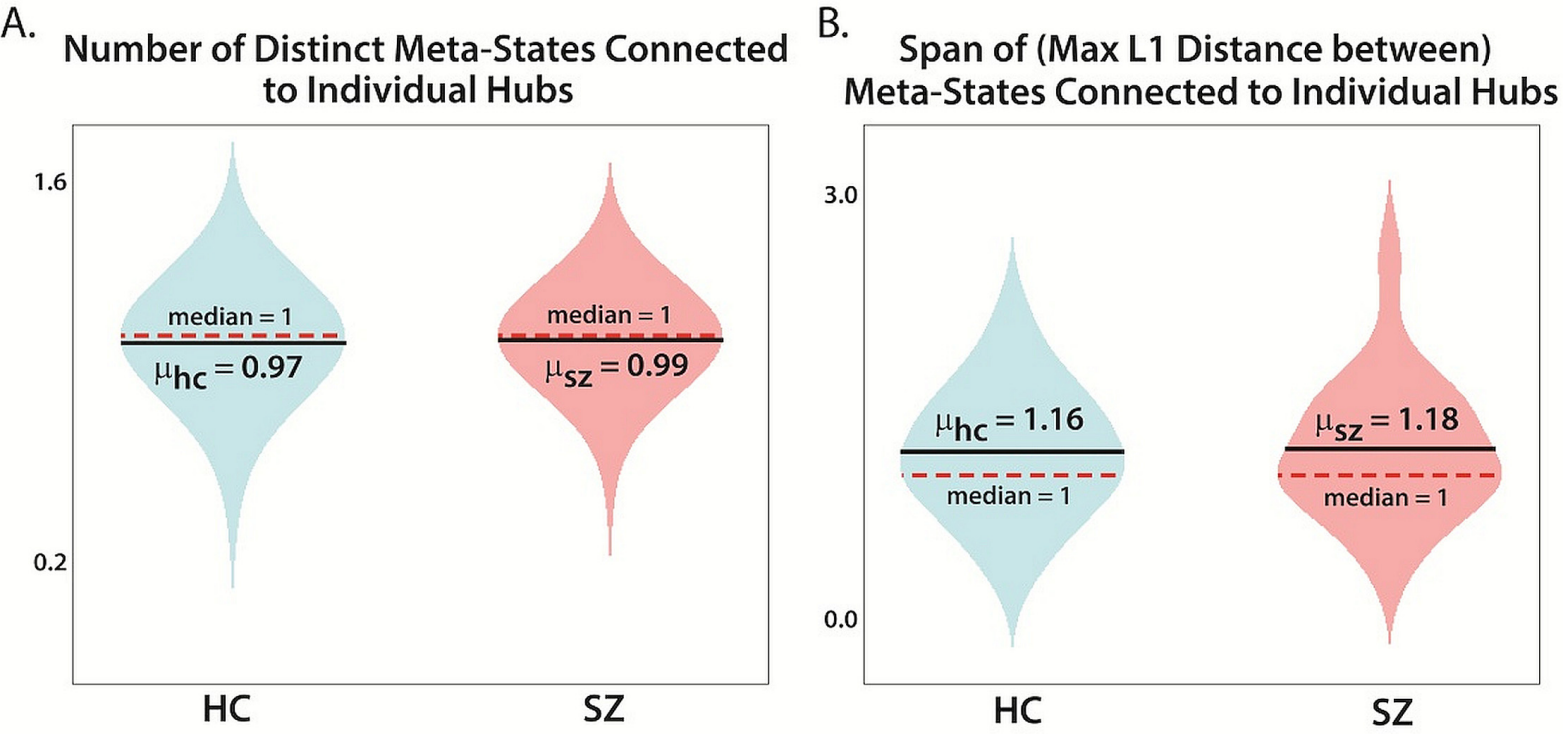
Dynamism measures restricted to immediate neighborhoods of higher-level hubs. We examine the number and the span of incoming and outgoing meta-states connected to each hub of level 8 and over. The interval was selected so that more than a third of HCs and of SZs have level-k hubs for k≥the lower bound of the interval. The values are not integers because we average over each segment during which a given hub is occupied for a subject, ie. the subject first visits some level k hub for 3 consecutive timepoints and then later visits the same hub again for k-3 consecutive timepoints. Each separate occupancy has its own outgoing target, which might or might not be identical. Since the number of distinct segments of occupancy of a given hub affect the overall number and range of the targets of that hub, we rescale by $\frac{1}{number\;of\;segments}$ resulting in non-integer valued measures. (A) The number (rescaled as indicated) of distinct target meta-states of fixed hubs; (B) The span (maximal L1 distance between, also rescaled as indicated above) of target meta-states of fixed hubs.

Simultaneously considering these results and combining their implications, what emerges is a picture of generally more cautious, sluggish patient trajectories through constrained regions of connectivity space, contrasted with the relatively quick, fluid, more sprawling trajectories of healthy subjects. Deeper investigations of meta-state occupancy, recurrence, span and change patterns however reveal a richer and more nuanced perspective on the distinctive ways patients and controls move dynamically through connectivity space. Schizophrenia patients, for example, tend to have a larger number of relatively more absorbing hub states than controls. Patients travel less distance through the state space from one time point to the next on average, and the largest *k*≥*4* for which patients have a level-*k* hub tends to exceed that for healthy controls. Although not statistically significant, it is also the case that the directional effect of schizophrenia diagnosis on the number and span of states that are the outgoing targets of hubs is positive (Fig 13). Locally, in the immediate neighborhood of hubs, the schizophrenia patients seem slightly more dynamic than controls, while globally they are significantly less so.

Although both groups have hub states that they return to relatively often, any routing role these hubs play is significantly less efficient in patients than controls and also somewhat more erratic: patients have more hubs of each level, their hubs hold incoming trajectories longer, and ultimately release trajectories to a slightly wider range of targets. We see an analogous scaling phenomenon when we make an upward adjustment to the number of visits to a state required for it to qualify as having been occupied. If the threshold is one visit, then controls visit significantly more states. When the threshold is shifted upward to four or more visits, then it is patients that are sampling a larger number of states (Fig 10). Finally, although patients are individually occupying fewer states and moving smaller distances in the state space, their trajectories are nonetheless more likely to intersect at some meta-state than are trajectories of healthy subjects (Fig 12(B)) and the set of distinct meta-states that are in the joint intersection of some collection of patients is almost always (except in the case of pairs of subjects) larger than the set of points that sit in the joint intersection of a similarly sized collection of controls (Fig 12(A)).

**Fig 14:**
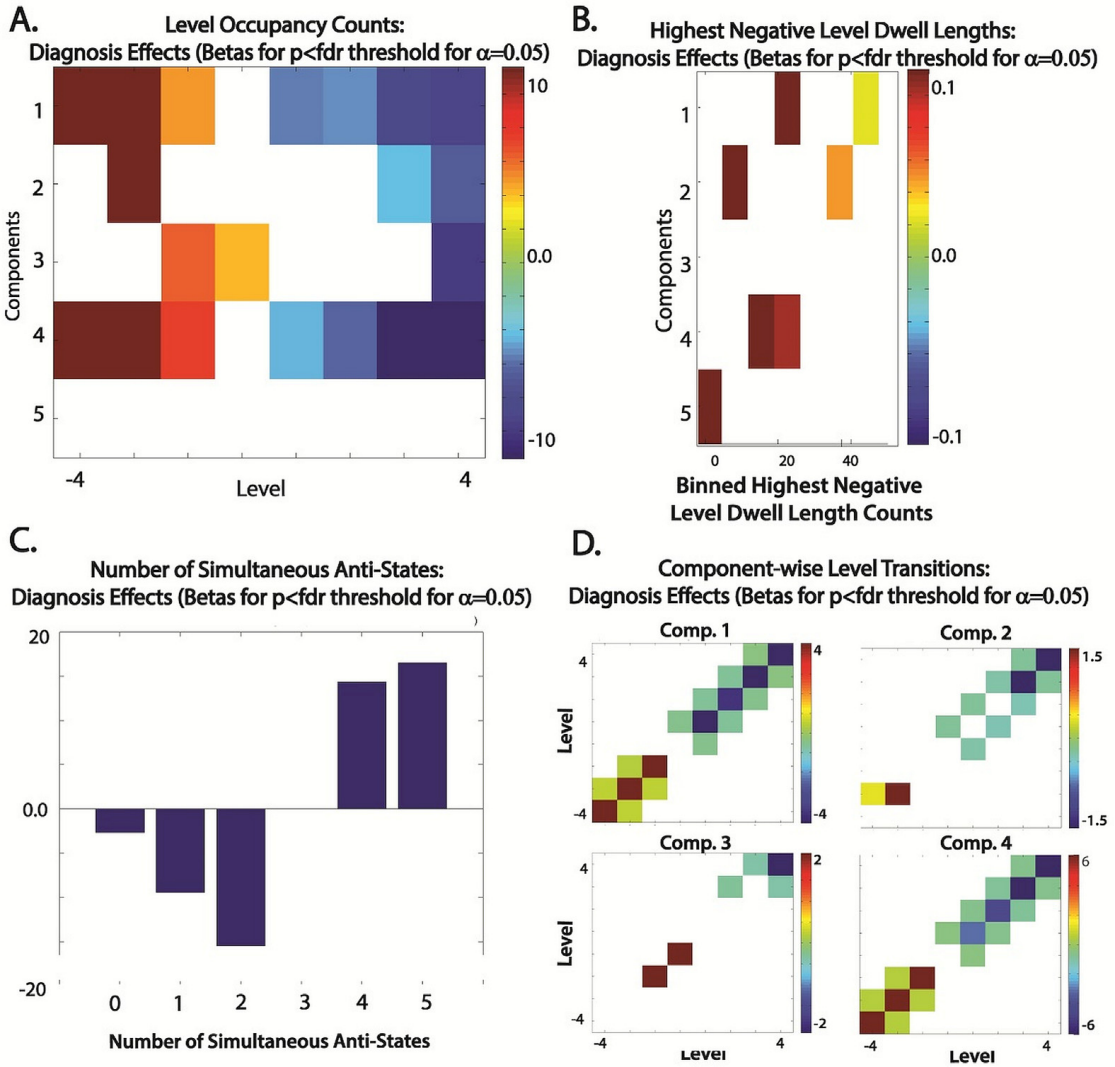
Effect of Schizophrenia on Dynamic Roles of Individual tICA Correlation Patterns (Fig 4(A), Row 1). Red (or bars pointing upward) indicate positive correlation with SZ. Only effects significant at the 0.05 level following FDR-correction are displayed. (A) SZ effects on the number of times each discretized CP timecourse (y-axis), assumes values indicated on the x-axis; (B) SZ effects on binned counts (x-axis) of the number of timepoints each discretized CP timecourse spends *consecutively* the most negative level, -4; (C) SZ effect on the number of correlation patterns *simultaneously* contributing in their *anti-state* form, ie. on the number of timepoints at which a subject's meta-state contains the indicated number (x-axis) of negative values; (D) SZ effects, component-wise for CPs that exhibit strong positive AVSN correlations, on the number of transitions between levels indicated on x-axis and y-axis; All diagnosis effects and p-values are from the regression model specified in the Methods section.

Our findings demonstrate the flexibility, sensitivity, and robustness of the meta-state framework for dynamical analysis proposed here. Schizophrenia is a complex illness that presents with enormous variation of symptom combinations and intensities, and its effects on resting fMRI are commensurately complex. Most studies to date have taken a static point of view, assuming the characteristic of interest to be stationary on the timescale of the scan. Such studies have yielded consistent evidence that at both voxel and network levels that the signals of schizophrenia patients contain more high frequency content than healthy controls [1, 47]. Supporting the so-called *dysconnectivity hypothesis* [48] about schizophrenia, static functional network connectivity analyses have consistently revealed diminished network connectivity strengths in schizophrenia patients [7, 49–53], and more specifically altered connectivity between auditory, visual and somatosensory networks (AVSNs) and also between subcortical (SC) networks and AVSNs [26, 37]. Many of these findings appear to hold even when the stationarity assumption is lifted as evidenced by significantly more negative SZ timecourse values (Fig 14) for CPs that feature strong positive intra-AVSN correlations and strong negative SC-to-AVSN connections (Fig 4(A), Top row).

**Fig 15:**
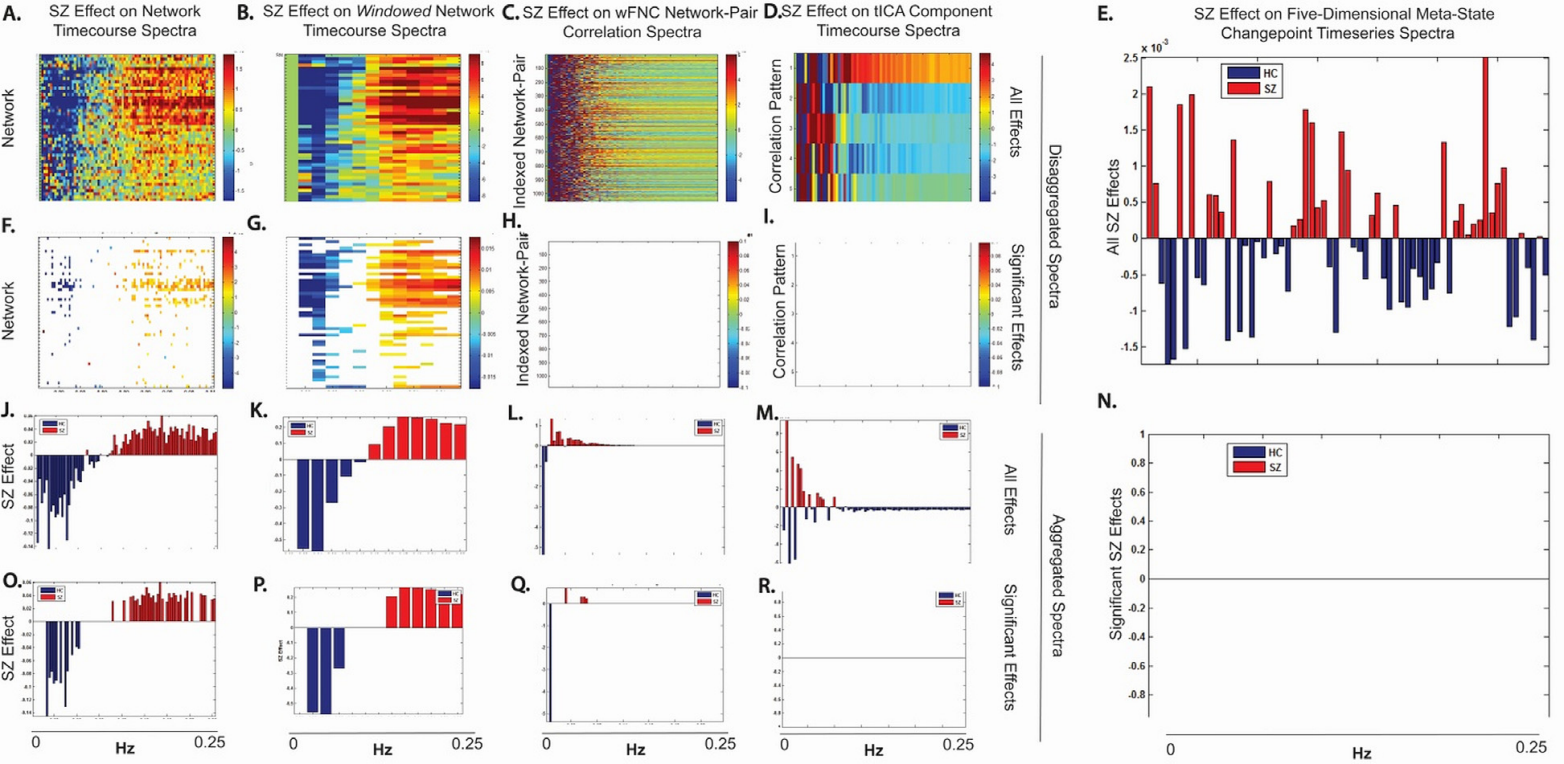
Effect of Schizophrenia on Spectral Power as Data is Transformed from Voxels to Networks to Meta-States SZ effects on the spectral power for inputs (Columns 1–4) to the meta-state dynamic connectivity framework and its output (Column 5) (red indicates positive correlation with SZ); (Column 1) (A) SZ effects on network TC spectra; (F) Effects from (A) that survive FDR correction at 0.05 significance level; (J) SZ effect on average spectral power over all network TCs; (O) Effects from (J) that survive FDR-correction at 0.05 significance level; (Column 2) (B) SZ effect on windowed network TC spectra; (G) Effects from (B) that survive FDR-correction at 0.05 significance level; (K) SZ effect on the average spectral power over all windowed network TCs; (P) Effects from (K) that survive FDR correction at the 0.05 significance level; (Column 3) (C) SZ effects on wFNC network-pair correlation spectra; (H) Effects from (C) that survive FDR-correction at the 0.05 significance level; (L) SZ effect on average spectral power over all wFNC network-pair correlations; (Q) Effects from (L) that survive FDR-correction at the 0.05 significance level; (D) SZ effects on tICA correlation pattern TCs; (I) Effects from (D) that survive FDR-correction at the 0.05 significance level; (M) SZ effect on average spectral power over all tICA correlation pattern TCs; (R) Effects from (M) that survive FDR-correction at the 0.05 significance level; (E) SZ effect on spectral power of the timeseries that is '1' when a five-dimensional meta-state changes to a different meta-state and '0' otherwise; (N) Effects from (E) that survive FDR-correction at the 0.05 significance level; All diagnosis effects and p-values are from the regression model specified in the Methods section.

For example, the window-wise spectrum of windowed network timecourses in schizophrenia patients have more high frequency content than those of healthy controls (Fig 15). In the timecourses of individual wFNC network-pair correlations, schizophrenia patients also exhibit greater high frequency power, though the effect is weaker statistically than for network and voxel timecourses (Fig 15, first three columns). Shen et al [54] have reported mixed effects of SZ on low-frequency power in windowed network-pair correlation timeseries, with patients exhibiting less low frequency power than controls in DMN-to-sensorimotor and DMN-to-cerebellum connections. EEG microstate studies [55–58] that identify common patterns of millisecond-scale activation from spatially segmented brain regions also indicate a higher rate of pattern-shifting among schizophrenia patients. In contrast, our investigation of whole-brain *ensembles* of network-pair correlations has yielded highly robust findings of *diminished* connectivity dynamism for schizophrenia patients. These findings are consistent with recent results on dynamic graph metrics, which inherently involve ensembles of nodal connections [59] and with the disappearance of positive high-frequency spectral SZ effects on ensemble-scale timeseries (Fig 15, last two columns). The results we present are fundamentally different in kind from those obtained in static settings. Our work concerns the adaptive fluidity and range of functional brain connectivity at a broad multi-network scale–both intrinsically dynamic phenomena that do not have static analogues. In future work however we plan to investigate connections between these high-level dynamic features that suggest group differences in connectonomic adaptivity and the underlying strength, modularity and other key characteristics of static and structural connectomes. It seems possible, for example that the consistent findings [1, 7, 48–53] of diminished connectivity strength and weakened connectome-wide modularity among schizophrenia patients reflect a degradation of communication pathways that enable rapid and widespread reorganization of network relationships.

The work we present adds an important new layer to the growing constellation of robust findings specific to certain scales or forms of analysis that may, eventually combine to produce powerful predictive fMRI signatures for this highly complex disease.
